# Macrophage frequency in the bone marrow correlates with morphologic subtype of myeloproliferative neoplasm

**DOI:** 10.1007/s00277-020-04304-y

**Published:** 2020-10-26

**Authors:** David C. A. Molitor, Peter Boor, Andreas Buness, Rebekka K. Schneider, Lino L. Teichmann, Ruth-Miriam Körber, Gabor L. Horvath, Steffen Koschmieder, Ines Gütgemann

**Affiliations:** 1 Institute of Pathology, University Hospital Bonn, Bonn, Germany; 2 Institute of Pathology, University Hospital Aachen, RWTH Aachen, Bonn, Germany; 3 Institute for Medical Biometry, Informatics and Epidemiology, Medical Faculty, University of Bonn, Venusberg-Campus 1, 53127 Bonn, Germany; 4 Institute for Genomic Statistics and Bioinformatics, Medical Faculty, University of Bonn, Venusberg-Campus 1, 53127 Bonn, Germany; 5 Department of Hematology, Erasmus MC Cancer Center, Rotterdam, Netherlands; 6 Institute for Biomedical Engineering Department of Cell Biology , RWTH , Aachen, Germany; 7 Department of Hematology and Oncology, University Hospital Bonn, Bonn, Germany; 8 Medical Faculty, Microscopy Core Facility, University of Bonn, Bonn, Germany; 9 Department of Hematology, Oncology, Hemostaseology, and Stem Cell Transplantation, Faculty of Medicine, RWTH Aachen, Aachen, Germany

**Keywords:** Myeloproliferative neoplasm, Macrophages, Bone marrow fibrosis, Immunohistochemistry

## Abstract

Bone marrow (BM) fibrosis in myeloproliferative neoplasms (MPNs) is associated with a poor prognosis. The development of myelofibrosis and differentiation of mesenchymal stromal cells to profibrotic myofibroblasts depends on macrophages. Here, we compared macrophage frequencies in BM biopsies of MPN patients and controls (patients with non-neoplastic processes), including primary myelofibrosis (PMF, *n* = 18), essential thrombocythemia (ET, *n* = 14), polycythemia vera (PV, *n* = 12), and Philadelphia chromosome–positive chronic myeloid leukemia (CML, *n* = 9). In PMF, CD68-positive macrophages were greatly increased compared to CML (*p* = 0.017) and control BM (*p* < 0.001). Similar findings were observed by CD163 staining (PMF vs. CML: *p* = 0.017; PMF vs. control: *p* < 0.001). Moreover, CD68-positive macrophages were increased in PV compared with ET (*p* = 0.009) and reactive cases (*p* < 0.001). PMF had higher frequencies of macrophages than PV (CD68: *p* < 0.001; CD163: *p* < 0.001) and ET (CD68: *p* < 0.001; CD163: *p* < 0.001). CD163 and CD68 were often co-expressed in macrophages with stellate morphology in Philadelphia chromosome–negative MPN, resulting in a sponge-like reticular network that may be a key regulator of unbalanced hematopoiesis in the BM space and may explain differences in cellularity and clinical course.

## Introduction

Classical myeloproliferative neoplasms (MPNs) are a heterogenous group of diseases arising from the bone marrow (BM), comprising Philadelphia chromosome (Ph)–positive chronic myeloid leukemia (CML) and the three Ph-negative (Ph−) MPN polycythemia vera (PV), essential thrombocythemia (ET), and primary myelofibrosis (PMF). While the Philadelphia-chromosomal BCR-ABL1 translocation is driving predominantly myeloid hyperplasia in CML, Ph− MPNs are driven by JAK-STAT signaling which is upregulated by different mutations (JAK2V617F, CALR, or MPLW515) and is a key event in the disease course [1, 2]. Enhanced JAK-STAT signaling leads to the release of proinflammatory and profibrotic cytokines [3] and chronic inflammation is considered as a major promoter of Ph− MPNs [4, 5]. Differences in heterozygosity of Jak2 mutations in Ph− MPNs explain only in part the predominance of hyperplasia in the erythroid lineage (PV), myeloid plus megakaryocytic (PMF), and megakaryocytic (ET) lineage, suggesting the existence of other disease-modifying factors.

Myelofibrosis in MPNs may result from increased cytokine production leading to activation of mesenchymal stromal cells (MSC) [6]. MSCs respond to higher levels of profibrotic cytokines by differentiation into myofibroblasts. The most important producers of profibrotic cytokines are megakaryocytes and macrophages [7].

Macrophages in the BM of MPN patients have been shown to be attractive novel cellular therapeutic targets, as they were shown to induce proliferation of myofibroblasts via vitamin D receptor signaling [8]. Interestingly, monocytosis confers a poor prognosis in PMF [9, 10] and monocyte-derived fibrocytes can be successfully inhibited in vivo by administration of the fibrocyte inhibitor serum amyloid P (SAP; pentraxin-2) [11].

Macrophages are increased in BM biopsies of PMF patients [12, 13]. Importantly, treatment with the Jak1/2 inhibitor ruxolitinib results in morphologic remission, eventually a decrease of fiber density as well as a decrease in M2-type macrophages and mast cells in the BM in approximately half of PMF patients [13, 14].

Anti-inflammatory M2 macrophages express the scavenger receptor CD163, which is upregulated by inflammatory cytokines such as IL-6 and IL-10 [15]. CD163 serves as a surrogate M2 marker by immunohistochemistry (IHC). However, it is unclear whether CD163 is a M2-specific marker of macrophages in the BM.

While macrophages have been shown to be increased in PMF [12], a direct comparison of macrophage frequency between the various MPN subtypes has not been performed. The goal of this study was to analyze the frequency and morphology of M2 macrophages in trephine biopsies of patients with Ph+ and Ph− MPNs preceding treatment.

## Methods and materials

### Patient cohort

BM biopsies from 61 patients were collected from 2003 to 2020 according to ethic board approval no. 235, University of Bonn. Each patient was diagnosed with ET, PV, PMF, and CML according to the WHO criteria [16] or underwent BM biopsy for other reasons without showing pathological features in the biopsy (see Table 1 for a detailed description of the patient cohort). Bone marrow biopsies with an MPN diagnosis at initial presentation were identified from the digital archive of the University of Bonn Hospital. Accelerated, blast phase, or pre-treated MPN cases were excluded from analysis. Only chronic phase CML cases were selected.

**Table 1:** Clinico-pathologic parameters and diagnoses, patient cohort. Bone marrow (BM) biopsies at initial diagnosis of patients with primary myelofibrosis (PMF), polycythemia vera (PV), essential thrombocythemia (ET) and chronic myeloid leukemia (CML) including 44 cases of Ph− MPN and 9 cases of Ph+ MPN (CML). Diagnosis and myelofibrosis grade (MF) are according to the current WHO criteria [16]. Percentages are in parentheses

Diagnosis	PMF	PV	ET	CML	Control	Total
Number of cases, *N* (%)	18 (30)	12 (20)	14 (23)	9 (15)	8 (13)	61 (100)
Median age (range)	63 (40–82)	59.5 (17–77)	64.5 (21–81)	58 (31–78)	59 (22–81)	61 (17–82)
Gender (%)						
Male	11 (61)	6 (50)	6 (43)	5 (46)	5 (63)	33 (54)
Female	7 (39)	6 (50)	8 (57)	4 (44)	3 (37)	28 (46)
MF grade (%)						
0	1 (6)	5 (42)	13 (93)	1(11)	7 (88)	27 (44)
1	10 (56)	5 (42)	1 (7)	8 (89)	1 (13)	25 (41)
2	2 (11)	2 (17)	0 (0)	0 (0)	0 (0)	4 (7)
3	5 (28)	0 (0)	0 (0)	0 (0)	0 (0)	5 (8)
Mutation (%)						
BCR-ABL1	0 (0)	0 (0)	0 (0)	9(100)	0 (0)	4 (15)
JAK2V617F	15 (83)	11 (92)	12 (86)	0 (0)	0 (0)	19 (73)
CALR	2 (11)	1 (8)	2 (14)	0 (0)	0 (0)	2 (8)
MPLW515	1 (6)	0 (0)	0 (0)	0 (0)	0 (0)	1 (4)
Mean hemoglobin [g/dl] (range)	12.2 (7.7–15.8)	16.2 (14.2–19.5)	14.0 (11.6–16.3)	10.8 (7.3–14.2)	11.6 (9.0–14.1)	13.0 (7.3–19.5)
Mean platelets/nl (range)	692.9 (95–2023)	477.3 (236–1107)	862.2 (600–1465)	232 (27–374)	108 (82–618)	566.1 (27–2023)
Mean WBC/nl (range)	14.3 (2.1–36.2)	15.6 (4.9–32.5)	10 (4.1–20.0)	146.5 (47.22–368)	9.8 (3.52–14.2)	33.0 (2.1–368)

### Immunohistochemistry and scoring

Standard morphology was assessed using H&E-stained slides. For immunohistochemistry (IHC) staining, standard paraffin sections (2–3 μm) were dried at 65 °C. Slides were then placed into retrieval solution (pH 6.0, Medac PMB-1-250). Afterwards, sections were washed with washing buffer (Medac B1-30A), then with distilled water. Endogenous peroxidase was blocked using H_2_O_2_. IHC was performed with primary antibodies against CD163 (clone MRQ-26, Medac, 1:1000) and CD68 (clone PGM1, Agilent, 1:100) and developed using a DAB IHC detection system on a semi-automatic immunohistochemistry stainer (Autostainer 480S; Medac, Germany). Photomicrographs were taken with a BX51 microscope (Olympus, Germany) and a Zeiss AxioCam MRc5 camera using the Axiovision software (Carl Zeiss, Germany).

To quantify the number of CD68- and CD163-positive cells in IHC sections, the slides were scanned using the Mirax scanning system (3D Histecj. Hungary). CD68- or CD163-positive cells per all nucleated cells within all interpretable bone marrow spaces excluding hemorrhaged, bony, or crushed areas on a tissue section were analyzed using QuPath [17], an open-source software for pathologic image analysis. Due to the differences in bone marrow structure and depending on the fibrosis grade and frequency of vacuoles, we chose to enumerate IHC-positive cells per all nucleated cells detected by hematoxylin staining as a marker for macrophage frequency. QuPath was used for detection of CD68- and CD163-immunopositive cells with hematoxylin for nuclear detection. Cell detection for images was performed with the following default settings: requested pixel size was set to 0.5 μm; for the nucleus, the background radius was set to 8 μm; the median filter radius was set to 0 μm; the intensity threshold was set to 0.2 for the cell: DAB OD mean scoring compartment. The total number of positive cells varied between 0 and 43% for CD68 and between 0 and 62% for CD163 in each image depending on cell frequency and marker expression. Results were verified using a semiquantitative scoring system (+, ++, +++) by two independent investigators (D.M., I.G.) showing similar results as the automatic counting using QuPath (data not shown).

### Confocal multiplex microscopy

Immunofluorescence multiplex staining was performed with Opal 7-Color manual IHC kit (AKOYA Biosciences: NEL811001KT). Formalin-fixed paraffin-embedded (FFPE) tissue blocks from bone samples were cut in 2-μm-thick sections. Slides were deparaffinized and antigen retrieval was performed in citrate buffer (EnVision FLEX target retrieval solution low pH, from Agilent: K8005) and the pT-Link (Agilent). After fixation in 4% formalin for 10 min, slides were washed and blocking was performed with H2O2 (DAKO real peroxidase blocking solution, Agilent: S2023) followed by 30-min incubation with antibody diluent (DAKO real antibody diluent, Agilent: S2022). The slides were then incubated with the first primary antibody CD68 (Agilent: M0876, dilution 1:2000) for 1 h in a humidified chamber at room temperature followed by detection with FLEX HRP (EnVision FLEX HRP, Agilent: SM802) and Opal 690 TSA Plus (AKOYA Biosciences, dilution 1:50), after which the slide was placed in citrate buffer (pH 6.0) and heated using microwave treatment (MWT). The slides were then incubated with the second primary antibody CD163 (Cellmarque: 163M-17, dilution 1:50) for 1 h in a humidified chamber at room temperature, followed by detection using the FLEX HRP and Opal 650 TSA Plus (AKOYA Biosciences, dilution 1:50), followed by incubation in citrate buffer (pH 6.0) with MWT. Counterstaining of cell nuclei was performed using Spectral DAPI (AKOYA Biosciences) and the sections were embedded with mounting medium (Vectashield Hard Set, Vector Laboratories: H-1400). For analysis, whole multiplex stained bone sections were automatically scanned with the Vectra 3.0 Automated Quantitative Pathology Imaging System (AKOYA Biosciences) (see Fig. 4 for results). Quantification of CD68 single-, CD163 single-, and CD68/CD163 double-positive cells was performed using CellProfiler [18].

### Statistical analysis

Statistical analysis was performed with the IBM SPSS (Chicago, IL) software package for Windows (version 25.0) and Microsoft Office Excel 365.

The Kruskal-Wallis test was used to compare the distribution of CD68- and CD163-positive cell frequency. Pair-wise comparison of diagnoses with respect to CD68 and CD163 frequencies was performed using Mann-Whitney *U* tests. Multiple testing correction for all comparisons was performed using a false discovery rate (FDR) (Benjamini-Hochberg procedure). Spearman rank and chi-square testing were used for pair-wise assessment of correlation between myelofibrosis (MF) grade, Hb, WBC, platelet count, and macrophage frequencies. Again, the multiple testing error for all comparisons was addressed by the Benjamini-Hochberg procedure. The FDR value is reported next to the *p* values in the “Results” part and figure legends.

## Results

Histological, clinical, and molecular characteristics of a patient cohort composed of a total of 44 cases of Ph− MPN (PMF (18), PV (12), ET (14)) and nine cases of Ph+ MPN (CML (9)) as well as eight reactive control BM have been summarized in Table 1.

**Fig. 1:**
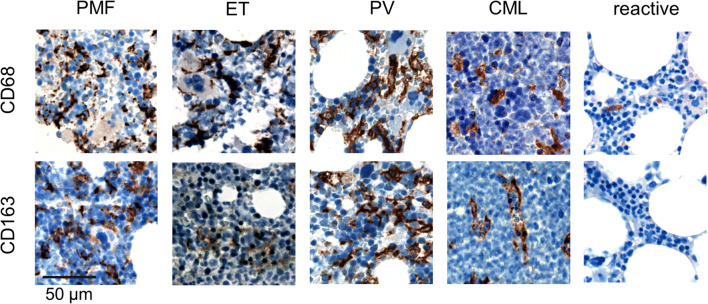
Macrophage frequency and distribution in MPN and reactive bone marrow biopsies. Representative photomicrographs showing CD68-expressing macrophages by IHC (upper) and CD163 staining (lower). Percentages of CD68-positive cells determined by automatic cell counting in the upper row cases from left to right: PMF (30%), ET (2%), PV (18%), CML (2%), and control (2%). Percentages of CD163-positive cells in the lower row paired samples from left to right: PMF (38%), ET (0%), PV (23%), CML (2%), and control (2%). Evenly dispersed CD68- and CD163-expressing macrophages, with stellate morphology, are increased in Ph− MPN (especially in PMF and PV) and show a more ovaloid morphology in CML (× 400 magnification)

**Fig. 2:**
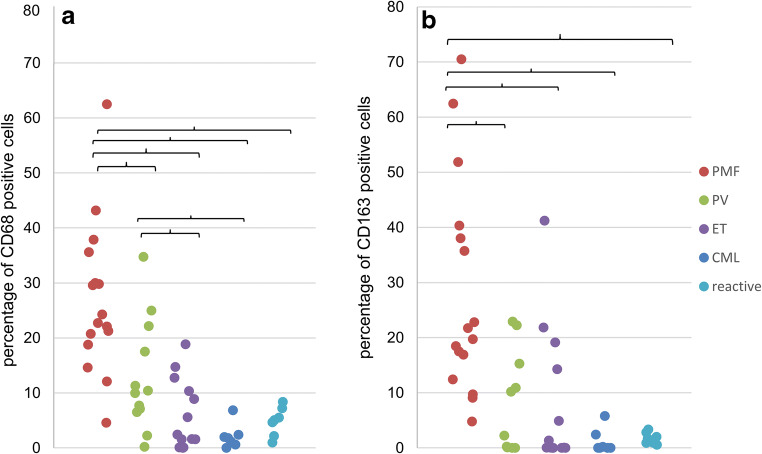
Correlation of macrophage frequency with type of MPN. Scatter plot, CD68 (A) and CD163 (B) frequencies in biopsies of MPN patients (PMF, PV, ET, CML) and reactive BM controls. CML and reactive BM contain few macrophages, while PMF biopsies contain higher frequencies of CD68- and CD163-positive macrophages (% per nucleated cells). PV was associated with a significant higher macrophage frequency than CML or ET. Brackets indicate significant differences between diagnostic subtypes (*p* < 0.05)

While in control BM and CML patients, macrophages were sparse and distributed evenly within the BM space, macrophages were increased significantly in PMF > PV > ET (Figs. 1 and 2).

**Fig. 3:**
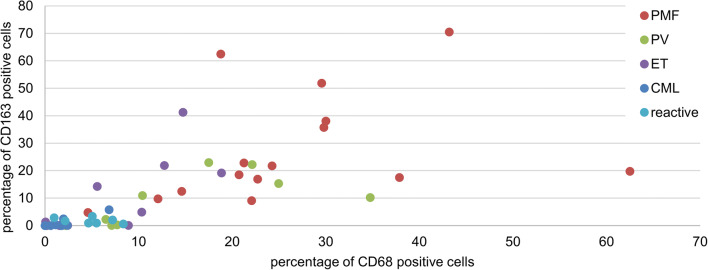
Correlation of CD68-positive and CD163-expressing macrophage frequency. Macrophage frequencies determined by CD68 and CD163 IHC staining (immunopositive cells per all nucleated cells, QuPath analysis)

Significantly higher CD68 frequencies were found in PMF BM (mean CD68-positive cells: 27% per all nuclear cells within bone marrow spaces) compared to CML (mean 2% CD68-positive cells) (*p* = 0.017, FDR = 0.028), ET (mean 6% CD68-positive cells) (*p* < 0.001, FDR = 0.008), PV (mean 13% CD68-positive cells) (*p* < 0.001, FDR = 0.015), and reactive BM (mean 5% CD68-positive cells) (*p* < 0.001, FDR = 0.003) (Figs. 2 and 3). Significant differences in CD163-positive macrophage frequencies were found between PMF (mean 28% CD163-positive cells) and CML (mean 1% CD163-positive cells) (*p* = 0.017, FDR = 0.025), PMF and ET (mean 7% CD163-positive cells) (*p* < 0.001, FDR = 0.005), PMF and PV (mean 8% CD163-positive cells) (*p* < 0.001, FDR = 0.013), and PMF compared with reactive BM (mean 2% CD163-positive cells) (*p* < 0.001, FDR = 0.010) (Fig. 2b). In addition, CD68 frequencies were higher in PV than in reactive (*p* < 0.001, FDR = 0.018) and higher in PV than in ET (*p* = 0.009, FDR = 0.023) (Fig. 2a).

Overall, a correlation of CD68 and CD163 expressions by IHC on serial sections was observed within the entire cohort (Fig. 3).

**Fig. 4:**
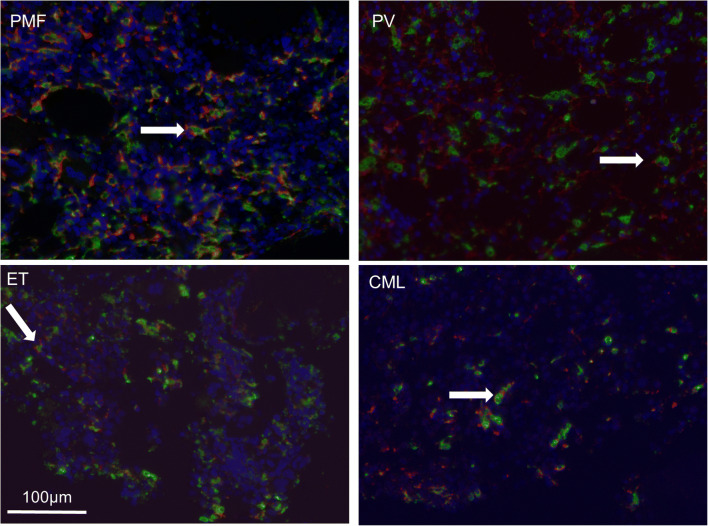
Immunofluorescence co-staining of CD68 and CD163 in MPN biopsies. Multispectral microscopy reveals frequent co-localization of CD68- (green) and CD163 (red)-positive macrophages, nuclei highlighted in DAPI (blue), representative images (× 200) (white arrow: CD68/CD163 double-positive macrophages)

Interestingly, in 13 out of 61 BM biopsies, CD163-positive cell frequencies were higher than CD68-positive macrophage frequencies in corresponding IHCs. We therefore determined whether CD68 and CD163 were co-expressed on identical cells using multiplex multispectral imaging confocal microscopy for selected cases. Indeed, both molecules were frequently co-expressed in cells with macrophage morphology, most prominently in PMF and PV (Fig. 4). Some macrophages were only positive by confocal microscopy with anti-CD163 or anti-CD68 (Fig. 4). Quantitative cell analysis using CellProfiler on selected cases showed the following percentages of CD68 and CD163 double-positive and single-positive macrophages per all nucleated cells: PMF: 60%, 62%, 53%; PV: 42%, 80%, 40%; ET: 56%, 70%, 52%; CML: 18%, 26%, 16% (percentage of CD68 single-positive, CD163 single-positive and double-positive cells respectively).

**Fig. 5:**
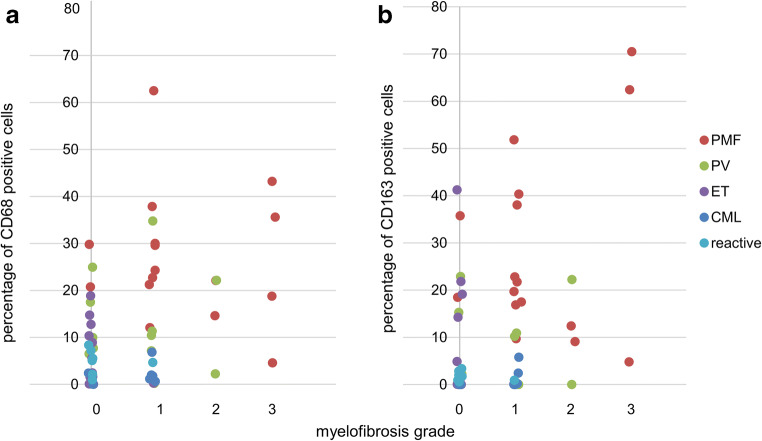
Myelofibrosis (MF) grade and macrophage frequency. Scatter plot comparing MF grade and macrophage frequency using CD68 (A) and CD163 (B) automatic cell counts (% positive cells by IHC per nucleated cells, QuPath analysis) in BM trephines. MF grade was determined using a four-tiered scale [16]: MF grade with no increase in reticulin fibers (grade 0), loose increase of reticulin (1), more diffuse and dense increase in reticulin and beginning collagen fibers (2), and dense reticulin and collagen fibrosis (3)

No correlation between CD68- or CD163-positive macrophage frequency and grade of myelofibrosis (MF) of all MPN cases combined or selected per diagnostic subtype was observed (Fig. 5).

However, when early PMF or ET (MF 0–1) were selectively analyzed, a significant difference of CD68- (*p* < 0.001) as well as CD163-positive cell frequencies (*p* < 0.001) could be demonstrated (PMF: mean 29% CD68-positive cells and mean 27% CD163-positive cells vs. ET: mean 7% CD68-positive cells and mean 8% CD163-positive cells/nucleated cells). However, in this subgroup, the majority of PMF cases showed mild diffuse myelofibrosis, whereas ET cases were mostly MF 0 (10 out of 11 PMF cases had MF grade 1 and 13 out of 14 ET cases had MF grade 0).

## Discussion

The above results underline the importance of macrophages in MPN-associated alterations of the immune tumor microenvironment (TME) and regulation of cell lineage turn over. Macrophages have intimate spatial contact and functional relationship with developing hematopoietic cells [19, 20].

We show that macrophage abundance differs in Ph+ MPN (CML) and Ph− MPN: PMF, PV, and ET with PMF have the highest frequency in BM biopsies, followed by PV and ET.

Previous work on macrophage frequency in MPN has shown that macrophages are far more frequently observed in BM biopsies than in BM aspirates [21, 22]. Based on our observations in situ as well as others, macrophages are intimately connected with the non-cellular and cellular BM stroma. In Ph− MPN, CD68- and CD163-positive macrophages demonstrated an irregular stellate shape with slender cytoplasmatic processes (Figs. 1 and 4) which may explain why these cells are difficult to aspirate, rendering functional analysis technically challenging. In contrast, in CML, macrophages had a more ovaloid appearance.

While macrophages in the BM are quite abundant (Figs. 1 and 4), it is currently unclear which fraction of these CD68- and CD163-positive macrophages is truly residing in a long-term quiescent state and which fraction is generated through differentiation from monocytes.

Our findings of differences in macrophage frequencies in various types of MPN extend previous results showing that macrophage and mast cell frequencies in BM biopsies normalize after Jak1/2 inhibition in a significant proportion of PMF patients accompanied by decreased grades of fibrosis [12, 13]. In the study of Kvasnicka et al., macrophages were reported to correlate with the degree of myelofibrosis in PMF, although a detailed presentation of the data was not provided [13].

While our cohort suffices to demonstrate differences of macrophage frequencies between subtypes of MPN (Fig. 3), this cohort is too small to analyze correlations of macrophage content and peripheral blood counts. Prospective clinical cohorts will need to determine a correlative or predictive value of CD163 or CD68 as a parameter accompanying or predicting myelofibrosis. Interestingly, macrophages are more abundant in PMF with none to mild myelofibrosis (MF grade 0–1) than in ET (MF grade 0–1) opening up the possibility for improved diagnostic separation of these entities in early myelofibrotic stages. Additional case cohorts are needed to confirm this data and in order to exclude fiber grade as a confounding variable in this subgroup analysis.

Macrophages in the BM are not only involved in hematopoietic stem cell homeostasis via regulation of Coxsackie and adenovirus receptor (CAR)–positive fibroblasts [23] but also in myeloid cell turnover, in particular in the elimination of short-lived granulocytes [24, 25]. Furthermore, macrophages regulate erythropoiesis within erythropoietic islands via turnover of expelled nuclei and secretion of erythropoietin [26]. Thus, one possible explanation for the increased frequency of macrophages in PMF > PV > ET may be the high turnover of individual cell lineages in these diseases with PMF being the disease with the most pronounced increase in granulopoiesis in the bone marrow.

Since molecular aberrations such as JAK2V617F, CALR, or MPL mutations are shared between Ph− MPN, it is currently unclear whether the high frequency of macrophages is a consequence or a cause of the phenotype (hyperplasia of selected cell types). The Jak2 mutation has been detected in monocytes [27]; thus, at this point, BM macrophages could be part of the malignant clone. Interestingly, M2 macrophages are centrally involved in fibrosis through secretion of profibrotic cytokines in PMF as well as in tissue regeneration and scar formation [28].

The upregulation of MAF (v-maf avian musculoaponeurotic fibrosarcoma oncogene homolog) in PMF CD34+ hematopoietic progenitor cells (HPCs) results in enhanced monocyte/macrophage and megakaryocyte differentiation as well as increased production of proinflammatory/profibrotic cytokines and growth factors (CCL2, IL8, MMP9, LGALS3, SPP1), leading to MSC proliferation and collagen production [29]. Given the proinflammatory nature of macrophages in PMF and our own findings, we suspect that CD163 is not a good marker of M2 polarization in PMF (Fig. 3). Furthermore, given that CD163+ macrophages are often more frequent than CD68+ macrophages (Figs. 1 and 4), it is unlikely that CD163 is a M2-specific marker provided that CD68 is a pan-macrophage marker in the BM.

Further in vitro and in vivo studies are needed to better characterize the function of tissue-resident macrophages in MPN with stellate morphology, keeping in mind that these cells are difficult to aspirate.

Macrophages in the BM of MPN patients have recently gained increased clinical attention, as they have been shown to be attractive novel cellular therapeutic targets, particularly as they play a significant role in inducing proliferating myofibroblasts via vitamin D receptor signaling [8].

Our findings support efforts to investigate macrophages as cellular targets in therapeutic trials of MPN patients further. Preclinical and clinical studies would be helpful to address whether targeting the monocyte/macrophage system, in addition to inhibition of JAK1/2, in first-line or advanced patients is of future therapeutic benefit.

## Data Availability

Raw data is available to any researcher wishing to use them for non-commercial purposes, without breaching participant confidentiality upon request (contact corresponding author).
